# High CD133 expression in proximal tubular cells in diabetic kidney disease: good or bad?

**DOI:** 10.1186/s12967-024-04950-0

**Published:** 2024-02-16

**Authors:** Yuhan Zhang, Lusi Xu, Congcong Guo, Xianzhi Li, Yutian Tian, Lin Liao, Jianjun Dong

**Affiliations:** 1 Department of Endocrinology and Metabology, The First Affiliated Hospital of Shandong First Medical University & Shandong Provincial Qianfoshan Hospital, Jinan, 250014 China; 2 Department of Endocrinology, Shandong Provincial Hospital Affiliated to Shandong First Medical University, Jinan, 250021 Shandong China; 3 Shandong Key Laboratory of Endocrinology and Lipid Metabolism, Jinan, 250021 Shandong China; 4 Division of Endocrinology, Department of Internal Medicine, Qilu Hospital of Shandong University, Jinan, 250012 China

**Keywords:** Diabetic kidney disease, CD133, Proximal tubular cells, Apoptosis, Data mining

## Abstract

**Background:**

Proximal tubular cells (PTCs) play a critical role in the progression of diabetic kidney disease (DKD). As one of important progenitor markers, CD133 was reported to indicate the regeneration of dedifferentiated PTCs in acute kidney disease. However, its role in chronic DKD is unclear. Therefore, we aimed to investigate the expression patterns and elucidate its functional significance of CD133 in DKD.

**Methods:**

Data mining was employed to illustrate the expression and molecular function of CD133 in PTCs in human DKD. Subsequently, rat models representing various stages of DKD progression were established. The expression of CD133 was confirmed in DKD rats, as well as in human PTCs (HK-2 cells) and rat PTCs (NRK-52E cells) exposed to high glucose. The immunofluorescence and flow cytometry techniques were utilized to determine the expression patterns of CD133, utilizing proliferative and injury indicators. After overexpression or knockdown of CD133 in HK-2 cells, the cell proliferation and apoptosis were detected by EdU assay, real-time cell analysis and flow analysis. Additionally, the evaluation of epithelial, progenitor cell, and apoptotic indices was performed through western blot and quantitative RT-PCR analyses.

**Results:**

The expression of CD133 was notably elevated in both human and rat PTCs in DKD, and this expression increased as DKD progressed. CD133 was found to be co-expressed with CD24, KIM-1, SOX9, and PCNA, suggesting that CD133+ cells were damaged and associated with proliferation. In terms of functionality, the knockdown of CD133 resulted in a significant reduction in proliferation and an increase in apoptosis in HK-2 cells compared to the high glucose stimulus group. Conversely, the overexpression of CD133 significantly mitigated high glucose-induced cell apoptosis, but had no impact on cellular proliferation. Furthermore, the Nephroseq database provided additional evidence to support the correlation between CD133 expression and the progression of DKD. Analysis of single-cell RNA-sequencing data revealed that CD133+ PTCs potentially play a role in the advancement of DKD through multiple mechanisms, including heat damage, cell microtubule stabilization, cell growth inhibition and tumor necrosis factor-mediated signaling pathway.

**Conclusion:**

Our study demonstrates that the upregulation of CD133 is linked to cellular proliferation and protects PTC from apoptosis in DKD and high glucose induced PTC injury. We propose that heightened CD133 expression may facilitate cellular self-protective responses during the initial stages of high glucose exposure. However, its sustained increase is associated with the pathological progression of DKD. In conclusion, CD133 exhibits dual roles in the advancement of DKD, necessitating further investigation.

**Supplementary Information:**

The online version contains supplementary material available at 10.1186/s12967-024-04950-0.

## Introduction

Diabetic kidney disease (DKD) is a typical microvascular complication of diabetes mellitus and the leading cause of end-stage renal disease [1, 2]. Proximal tubular cells (PTCs) are highly susceptible to damage due to high metabolic demands and play an important role in the pathogenesis of DKD [3]. Previous studies suggested that PTC lesions already exist at the early stage of DKD [4, 5]. Recent studies reported PTCs have significant regenerative potential. In acute kidney injury, PTCs undergo dedifferentiation characterized by the upregulation of injury markers and acquire similar characteristics to progenitor cells to participate in cell proliferation and renal repair [6–10]. These dedifferentiated cells express CD133, CD24, vimentin, and other progenitor markers [8, 11–13]. Additionally, kidney biopsies demonstrated that the CD133-positive cell population (CD133+ cells) was increased after renal insults in patients, indicating renal repair [13–15].

CD133 is a single-chain transmembrane glycoprotein encoded by the prominmin 1 gene (*PROM1*), and it is expressed on immature haematopoietic stem cells, tissue-specific progenitor cells, and cancer stem cells [16, 17]. In nonneoplastic kidney disease, CD133 is modulated by hypoxia and has been shown to promote cell proliferation and inhibit senescence [18, 19]. However, relevant studies have been limited to acute kidney disease, but not chronic kidney disease, especially in DKD.

In the present study, we analysed single-cell RNA sequencing (scRNA-seq) data of human early DKD combined with DKD models in vitro and in vivo, and described the expression patterns of CD133 in PTCs. In addition, we explored the molecular functions of CD133 in cell proliferation and apoptosis in high glucose (HG) induced PTCs injury and analysed the clinical relevance of CD133 expression in the progression of DKD. We found that CD133-positive PTCs (CD133+ PTCs) have characteristic molecular expressions of repair tendency, such as PCNA, CD24, vimentin, and SOX9. Though the increased expression of CD133 could protect PTCs from apoptosis, our data mining analysis suggested that these survival CD133+ PTCs or CD133 itself might involved in the pathogenesis of DKD by various mechanisms.

## Materials and methods

### Single-cell RNA-sequencing data analysis

The scRNA-seq raw datasets (GSE131882 [20]) were obtained from Gene Expression Omnibus (https://www.ncbi.nlm.nih.gov/geo/). We analysed the data using the Seurat 3.2.1 (https://cran.r-project.org/web/packages/Seurat/index.html). Low-quality cells were excluded if less than 500 or more than 6000, or if mitochondrial gene content for more than 5%; genes detected in less than five cells were removed. Before clustering, data were scaled and variables were regressed out by the ScaleData function in Seurat. Then, the RunPCA function and RunUMAP were employed for dimension reduction. Cell clustering assignment was performed via FindAllMarkers function and based on the marker genes provided by a previous study [21]. Then, the FeaturePlot function was used to visualize the gene expression profiles of CD133 in each cluster. Pearson’s correlation test was applied for the correlation relationship between each gene and CD133 in the PTC cluster. The top 200 positive co-expressed genes were mapped into the Functional Annotation tool of Database for Annotation, Visualization and Integrated Discovery (DAVID) 6.8 [22] (https://david.ncifcrf.gov/) to perform Gene Ontology (GO) biological processes (BP) analysis (p value < 0.05). We divided the cells of the PTC cluster into two sub-clusters according to whether CD133 was expressed in cell and visualized the differential genes in each sub-cluster using FindMarkers. The percentage of cells where the genes with significantly differential expression were collected to draw a heatmap with GraphPad Prism 7.0 (https://www.graphpad.com) and conduct GO_BP analysis (*p* value < 0.05).

### Animals

Male Sprague Dawley rats at 5–6 weeks old were randomly divided into the Sham group, the unilaterally nephrectomized (Unx) group, and the diabetic kidney disease (DKD) group (n = 30 per group). The Sham group received a sham operation without kidney damage [23]. To construct an accelerated diabetic nephropathy rat model, a single intraperitoneal injection of streptozocin (STZ; Solarbio, Beijing, China) at a dose of 45 mg/kg after 1 week of right nephrectomy [23]. While, the Unx group received the same nephrectomy, but were injected with an equal dose of citrate buffer. Three days after injection, blood glucose levels over 16.7 mmol/L were considered as diabetes. These three groups then were randomized into groups (n = 10 per group) and observed for either 4, 8, and 12 weeks. Blood samples were collected from the tail vein to evaluate blood glucose. At the study time-points, rats were transferred to metabolic cages individually to collect 24-h urine. Rats were sacrificed at the specified time and the left kidneys were rapidly removed, weighed, and snap-frozen in liquid nitrogen or fixed in 4% paraformaldehyde. In a subset of rats (n = 3 per group), tubular cells were isolated from fresh renal cortical tissue as described below. The animal experiments were approved by the Ethics Committee of Shandong University and followed the Guiding Principles for the Care and Use of Laboratory Animals of China.

### Biochemical, morphometry, and tubulointerstitial fibrotic analysis

Blood urea nitrogen (BUN) and serum creatinine (SCr) were detected by an automatic biochemistry analyzer (Chemray 800, Shenzhen, China), Urinary protein was measured with a urine protein assay kit (Jiancheng bioengineering, Nanjing, China), while urine microalbumin was determined by using an enzyme-linked immunosorbent assay kit (CUSABIO Engineering Co., Wuhan, China). The kidney index reflects kidney hypertrophy calculated by kidney weight/body weight (mg/g). The tissues were embedded in paraffin, and 3–4 mm thick sections were stained with PAS to demonstrate renal morphology and structure and mesangial matrix deposition. Tubulointerstitial fibrosis was assessed by Masson staining, and the extent of fibrosis was defined as the percentage of fibrotic regions relative to the total tissue area. The above quantitative analysis was conducted by Image-Pro Plus 6.0 (Media Cybernetics, Silver Spring, MD, United States).

### Immunohistochemistry

A standard immunohistochemical technique was performed on renal paraffin sections using antibody against CD133 (ab19898, Abcam, Cambridge, MA, USA) or NGAL (ab216462, Abcam) at 1:100 dilution [24]. The expression level of CD133 was assessed by the percentage of positive cells in the field of vision. Specifically, the percentage of CD133 positive cells was the number of positive cells/the number of nuclei × 100%. The expression level of NGAL was assessed by the ratio between the NGAL positive area and the total tissue area. The above quantitative analysis was conducted by Image-Pro Plus 6.0.

### Isolation of renal tubular cells

Renal tubular cells were isolated from fresh kidney cortex by mechanical trituration [25–27], to avoid affecting of glomerular CD133 expression. Briefly, the renal cortices were dissected and minced into small pieces with ophthalmic scissors in ice-cold phosphate buffer solution (PBS). Fluid was discarded, and a type II collagenase solution (1 mg/mL) was added. After digested at 37 °C for 30 min, tissues were filtered through an 80-mesh strainer and rinsed with PBS. The filtrates were further filtered through a 100 μm cell strainer (Biologix, Jinan, China). The renal tubular cells were harvested on the strainer, verified under a light microscope, and centrifuged for extraction of cell protein.

### Immunofluorescence analysis

Kidney tissues were fixed in 4% paraformaldehyde and dehydrated with 30% sucrose overnight. Then the samples were embedded in OCT (Sakura Finetek, Japan) and sectioned into 5–8 μm with a cryostat. Antigen repair buffer (Beyotime Biotechnology, Shanghai, China) was used for antigen repair. Following permeabilization, samples were incubated with primary antibodies overnight at 4 °C. The secondary antibodies were supplied at 37 °C for 1 h on the following day. For cell immunofluorescence, HK-2 cells and NRK-52E cells were seeded on slides in a six-well plate and incubated in specified treatment for 48 h. The slides were fixed with 4% paraformaldehyde and permeabilized. After this, cells and tissues were treated identically in subsequent steps. The primary antibodies include rabbit anti-CD133 (1:100), mouse anti-CD24 (1:100, sc-70600, Santa Cruz Biotechnology, Santa Cruz, CA, USA), mouse anti-aquaporin-1 (AQP1, 1:100, sc-32737, Santa Cruz Biotechnology), mouse anti-Vimentin (1:100, 60330-1-Ig, Proteintech, Wuhan, China), mouse anti-PCNA (1:100, 60097-1-Ig, Proteintech), goat anti-KIM-1 (1:100, AF3689, RD Systems, CA, USA). The following secondary antibodies were used: Donkey anti-mouse-Alex 488 (1:400, ab150109, Abcam), goat anti-rabbit-DyLight 488 (1:100, A23220, Abbkine, Wuhan, China), goat anti-rabbit-DyLight 594 (1:100, A23410, Abbkine), and donkey anti-goat-Alexa 647 (1:200, Invitrogen, CA, USA). Nuclear counterstaining was performed using DAPI. Immunofluorescence was visualized using Nikon A1R confocal microscope, and microscopic parameters were the following: 20× objective (Air, NA = 0.75, WD = 1.0 mm), 5× magnification, and the pixel size was 2.49 μm × 2.49 μm. Each immunofluorescence experiment is performed at a fixed z-position.

### Cell culture and treatment

The human proximal renal tubular cell line HK-2 (American TypeCell Collection, Rockville, MD, USA) was purchased from ProCell Corporation (Wuhan, China) and cultured in MEM medium supplemented with 10% fetal bovine serum (Gibco, USA) and 1% penicillin–streptomycin (HyClone, Logan, UT, USA). NRK-53E, a rat proximal renal tubular cell line, was obtained from ProCell Corporation and maintained in DMEM with 5% fetal bovine serum and 1% penicillin–streptomycin. All cells were incubated at 37 °C with 5% CO_2_. For high glucose (HG) treatment, six concentration gradients were set as 10 mM, 20 mM, 30 mM, 40 mM, 50 mM, and 60 mM, respectively. After a 48-h culture, the protein expression level of CD133 was detected to determine the optimal concentration. MEM medium containing 5.5 mM D-glucose was used as the normal glucose (NG) group. The osmotic pressure was adjusted with mannitol.

### CD133 knockdown and overexpression

HK-2 cells were plated in six-well plates at a density of 2 × 10^5^ cells overnight. Plasmid and small interfering RNA (siRNA) transfections were performed using Lipofectamine 3000 (Invitrogen, USA) according to the manufacturer protocol. The following siRNA sequences (GenePharma, Shanghai, China) were used in the study: sense-CUGGGAAGCUAUUUAAUAA, antisense-UUAUUAAAUAGCUUCCCAG (siCD133-1); sense-GGCUGCUGUUUAUUAUUCUTT, antisense-AGAAUAAUAAACAGCAGCCTT (siCD133-2); sense-GGGCUAUCAAUCCCUUAAUTT, antisense-AUUAAGGGAUUGAUAGCCCTT (siCD133-3); sense-UUCUCCGAACGUGUCACGUTT, antisense-ACGUGACACGUUCGGAGAATT (negative control, NC). CD133 overexpression plasmid and control plasmid were purchased from Sino Biological Inc. (HG15024-NF, CV020, Sino Biological Inc., Beijing, China).

### Western blotting

Isolated renal tubular cells of rats, various cultured HK-2 cells, and NRK-52E cells were lysed with radioimmunoprecipitation assay (RIPA) buffer (Beyotime) and separated by SDS-PAGE. Primary antibodies against CD133 (1:500, Abcam), Vimentin (1:1000, Proteintech), CK-18 (1:1000, Boster), PCNA (1:1000, Proteintech), P53 (1:1000, #2524S, Cell Signaling Technology, MA, USA), Bax (1:1000, AF0120, Affinity), caspase-3 (1:500, ab32351, Abcam), cleaved caspase-3 (1:500, BF0711, Affinity), β-actin (1:5000, TA-09, ZSBIO, Beijing, China) and β-tubulin (1:5000, T0023, Affinity, IL, USA) and conjugated secondary antibodies (1:10,000, ZB-2301, ZB-2305, ZSBIO) were used. All blots were cut prior to hybridization with antibodies during blotting. Signals of targeted proteins were detected by ECL detection Reagent (ED0015-B, Sparkjade, China). Protein expression levels were normalized to those of β-actin or β-tubulin.

### Quantitative RT-PCR

Total RNA was extracted from the cultured cells by RNAiso Plus (TaKaRa, Dalian, China) referring to the manufacturer’s protocol. Reverse transcription was performed by a Prime-Script RT reagent kit (TaKaRa). Levels of mRNA were analysed by quantitative RT-PCR using SYBR green PCR master mix (Vazyme, Nanjing, China). The 2^−ΔΔCt^ method was used to calculate the relative expression levels and fold difference. GAPDH and β-actin served as internal references. The primer sequences are provided in Table 1.

**Table 1:** Primer sequences

Gene name	Forward-sequence	Reverse-sequence
CD133 (human)	ACTCCTTTTCAGGAGGGCAG	CGCGGCTGTACCACATAGAG
Vimentin (human)	AGGCGAGGAGAGCAGGATTT	AGTGGGTATCAACCAGAGGGA
ZO-1 (human)	TCACGCAGTTACGAGCAAGT	TGAAGGTATCAGCGGAGGGA
CD133 (rat)	TGACTGAAGCCCCAAAGCAA	TTATTCTGCCTCCCAGCACG
Vimentin (rat)	TTCTCTGGCACGTCTTGACC	TCATACTGCTGGCGGACATC
ZO-1 (rat)	AACAGAGCCGAGCAGTTAGC	GCAACATCAGCAATCGGTCC

### Flow cytometry

HK-2 cells were treated in accordance with the particular experimental groups and harvested by centrifugation after trypsin–EDTA treatment. The cells then were washed twice with pre-cooled PBS containing 2% FBS and resuspended. For every 200 μL of cell suspension, 5 μL of APC conjugated CD24 (17-0247-41, Invitrogen) and/or PE-conjugated CD133 (12-1338-41, Invitrogen) was added and incubated at room temperature for 30 min in dark. After washing twice, cells were resuspended in 500 μL PBS and tested by flow cytometer. For the apoptosis assay, apoptotic cells were measured using the Annexin V/PI apoptosis detection kit (BD Pharmingen, USA). HK-2 cells were collected as described above. Following centrifugation, 100 μL of 1× binding buffer was added. After incubation with 5 μL of Annexin V-FITC and 5 μL of PI for 15 min, each tube of cells was added 400 μL binding buffer and tested by flow cytometer. Cells were counted using BD FACS Aria II (BD Biosciences) and the data was analysed by FlowJo 10.6.2.

### Proliferation assay

HK-2 cells were seeded in 96-well plates at 8000 cells/well and treated according to the grouping to detect cell proliferation. The real-time proliferation of cells was monitored by time-lapse phase-contrast imaging using the IncuCyte S3 Live-Cell Analysis System. Real-time microscopic images were taken at 2-h intervals from 48 h. IncuCyte Zoom software was used to integrate the cell confluence algorithm and construct proliferation growth curves. The EdU assay was also performed using an EdU assay kit (RiboBio, Nanjing, China) to test cell proliferation. Briefly, cells were incubated with EdU and stained with Hoechst 33342, and then visualized under a fluorescence microscope (Leica DMi8). The percentage of EdU-positive cells was calculated by Image-Pro Plus 6.0.

### Statistical analysis

All data are expressed as the means ± standard error of the mean (SEM) from at least three independent experiments. Statistical analysis was performed using SPSS Statistics 22.0 (IBM SPSS Statistics, Chicago, IL). Student’s t-test was used to compare the significance between the two groups. For multiple-group comparisons, a one-way ANOVA followed by post hoc analysis was used. The correlation analyses were performed using Pearson correlation. A *p* < 0.05 was considered to denote statistical significance.

## Results

### ScRNA-seq analysis of CD133 expression and function in PTCs

**Fig.1:**
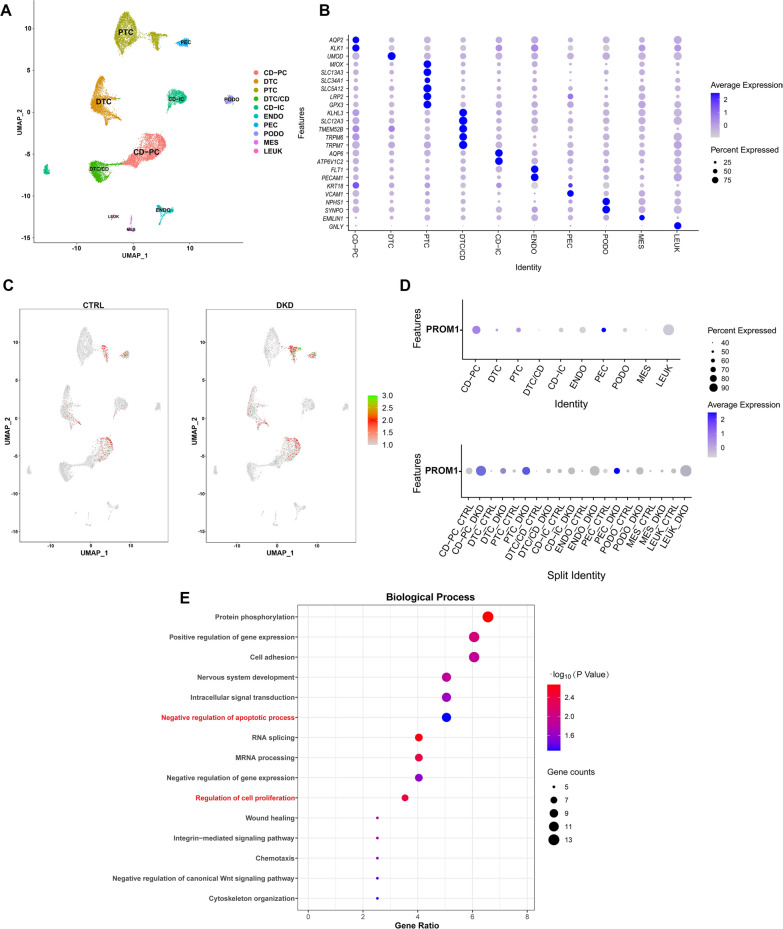
Analysis results of scRNA-seq of human DKD. **A** UMAP feature plot revealing the different clusters of renal cells. **B** Renal cell clusters were identified by specific marker expression. **C** Expression of CD133 (*PROM1*) in cell clusters in the DKD and the control (CTRL) groups. **D** Bubble plots showing the expression of CD133 (*PROM1*) in the combined data of DKD and CTRL, and split data. **E** GO_BP enrichment analysis of top 200 positive coexpression genes of CD133 (*PROM1*) in PTC cluster in combined data. *PTC* proximal tubular cell, *DTC* distal tubular cell, *CD-PC* collecting duct-principal cell, *DTC/CD* distal tubule cell/collecting duct, *CD-IC* collecting duct-intercalated cell, *ENDO* endotheliocyte, *PEC* parietal epithelial cell, *PODO* podocyte, *MES* mesangial cell, *LEUK* leukocyte

After quality control and data normalization, a total of 157,276 RNA features and 16,999 cells were used for downstream analysis. According to the differentially expressed genes in each cluster and canonical marker genes in a previous study [21], 10 clusters (Fig. 1A) were identified as: proximal tubular cell (PTC), distal tubular cell (DTC), collecting duct-principal cell (CD-PC), distal tubule cell/collecting duct (DTC/CD), collecting duct-intercalated cell (CD-IC), endotheliocyte (ENDO), parietal epithelial cell (PEC), podocyte (PODO), mesangial cell (MES), and leukocyte (LEUK). The expression of marker genes was described with a bubble diagram to display the characteristic genes of all cell clusters (Fig. 1B).

We next described and analysed the CD133 expression levels. UMAP feature plots (Fig. 1C) showed that CD133 (*PROM1*) was primarily clustered in PTC, PEC, DTC, and CD. CD133 expression was significantly increased in the PTC cluster in the DKD group compared to that in the control group (Fig. 1C, D). To further explore the molecular function of CD133, Pearson’s correlation analysis was used to compare other genes and CD133 in the PTC cluster to identify significantly positively coexpressed genes. The top 200 genes (Additional file 4: Table S1) were used to conduct GO_BP analysis and were enriched in 36 GO terms (Additional file 5: Table S2), and the top 15 terms are shown in Fig. 1E, which are associated with the regulation of gene expression, cell proliferation, apoptosis, cytoskeleton organization. These scRNA-seq results demonstrated the increased expression and possible molecular functions of CD133 in PTCs in early DKD.

### Establishment of the DKD models.

To verify and map the expression of CD133, we established DKD rat models at 4, 8, and 12 weeks, which allowed us to study changes of variable course in the kidney [28]. As shown in Additional file 6: Table S3, the body weights of rats in the DKD group were significantly reduced at each time point. In the DKD group, blood glucose levels increased gradually and reached a maximum value of the glucose meter (33.3 mmol/L) in the fourth week. Compared with those in the control group (the Sham and Unx groups), the kidney/body weight ratio, blood urea nitrogen (BUN), serum creatinine (Scr), urinary protein, and microalbumin levels in diabetic rats were significantly elevated from the fourth week and gradually increased over the time, indicating the progression of kidney damage (Additional file 1: Fig. S1A–C). Kidney histology and tubulointerstitial fibrosis were assessed by PAS staining and Masson staining (Additional file 1: Fig. S1D–F). In DKD rats, slight glomerular enlargement and tubule dilatation were observed in the fourth week. At week 8, DKD rats showed mild mesangial matrix hyperplasia, glomerular capillary thickening, and focal mild interstitial inflammatory infiltration. In addition to those lesions, mild interstitial fibrosis appeared at week 12. Furthermore, we performed immunohistochemical analysis using the NGAL antibody to evaluate the progression of renal tubular injury in DKD, and NGAL expression was distinctly increased at week 12 compared to that at week 4 and week 8 (Additional file 1: Fig. S1G).

### CD133 expression was increased in PTCs with the DKD progression

**Fig. 2:**
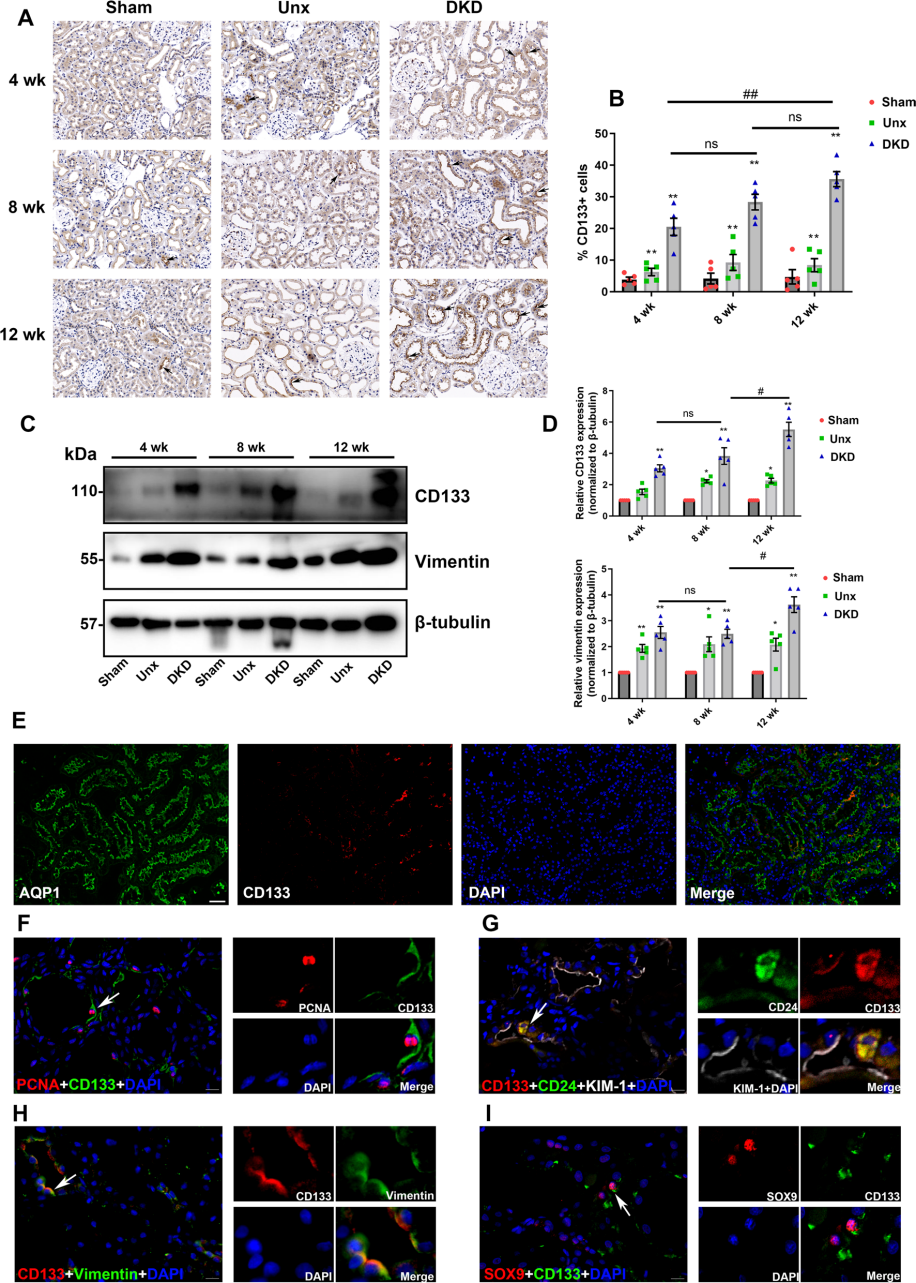
The expression patterns of CD133 in DKD rats. **A** The expression levels of CD133 were determined in different duration groups. The black arrows point to some of CD133-positive (CD133+) cells. Scale bar: 50 μm. **B** Semiquantitative analysis of CD133+ cells in kidney. **C**, **D** The protein expression levels of CD133 and vimentin in PTCs were detected by Western blotting. **E** Double-label immunofluorescence for CD133 (red) and aquaporin-1 (AQP1) (green) in rat kidney. Scale bar: 50 μm. **F** Double-label immunofluorescence for CD133 (green) and PCNA (red). **G** Triple-label immunofluorescence for CD133 (red), CD24 (green), and KIM-1 (white). White arrows indicate enlarged regions shown in the right panels. **H** Double-label immunofluorescence for CD133 (red) and vimentin (green). **I** Double-label immunofluorescence for CD133 (green) and SOX9 (red). Scale bar: 20 μm. Immunofluorescence staining was performed in 12-week DKD rats. *p < 0.05, **p < 0.01 versus the Sham group; ^#^*p* < 0.05, ^##^*p* < 0.01 versus the DKD group; ns: indicates nonsignificant

The expression of CD133 at different time points was examined by immunohistochemistry and western blotting. The results showed that the CD133 expression was increased in the renal cortical region in the DKD groups compared with the Sham group. Notably, the expression of CD133 in PTCs was significantly increased during the progression of DKD, and reached its highest level at 12 weeks (Fig. 2A, B). Moreover, to exclude the influence of other renal cells, PTCs were isolated and proteins were extracted to assess the expression level of CD133. Meanwhile, the expression level of Vimentin was evaluated, and the results showed that CD133 and Vimentin expressions were highest at 12 weeks in the DKD group (Fig. 2C, D). As shown in Fig. 2E, CD133 was expressed in AQP1 labeled PTCs, and mainly in the cytomembrane. Then, other immunofluorescence stainings were performed to describe the expression patterns of CD133 in kidney tissue of DKD using some indicators that were co-expressed with CD133 in acute kidney disease. Some fraction of CD133+ cells co-expressed PCNA, demonstrating that CD133+ cells with proliferative ability might be related to repair potential (Fig. 2F). The co-expression of CD133, CD24, and KIM-1 suggested that CD133 was expressed in damaged PTCs (Fig. 2G). Interestingly, results showed that CD133 and CD24 were mainly expressed in the cytoplasm. Moreover, CD133 was also co-expressed with Vimentin (Fig. 2H). According to previous studies [11, 29], these coexpressing cells were observed in acute kidney injury and also associated with renal repair. Another progenitor cell marker SOX9 was used to validate the progenitor characteristics of CD133+ cells, and results showed that some nuclei of CD133+ cells were SOX9-positive (Fig. 2I). These results indicated that CD133+ PTCs in DKD were similarly characterized and displayed to have similar expression patterns with those in acute kidney disease.

### High glucose induced the increased expression of CD133 in PTCs

**Fig. 3:**
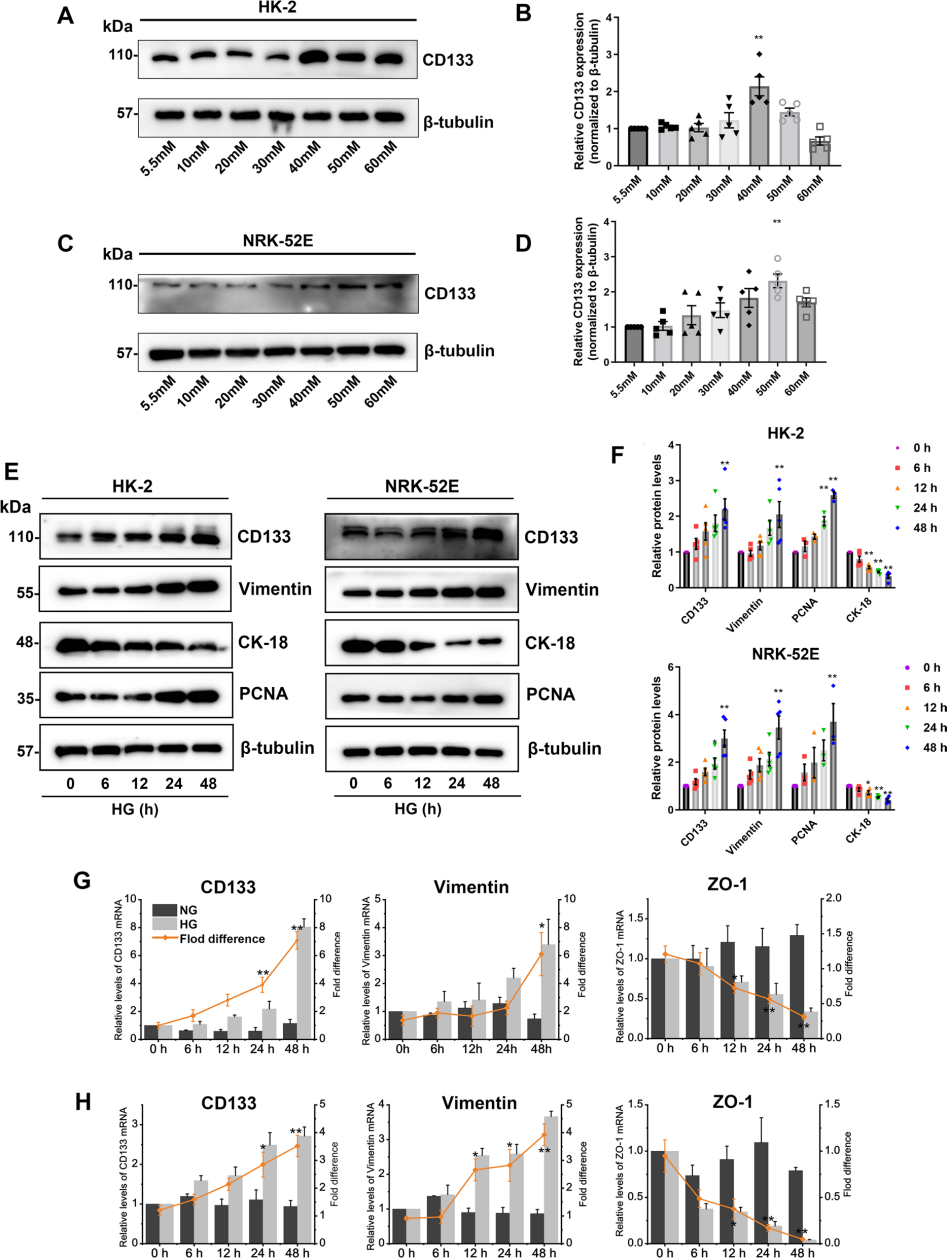
HG-induced the stem/progenitor and mesenchymal cell markers in HK-2 and NRK-52E proximal tubular cell lines. **A** Western blotting indicates the effect of various glucose concentrations on CD133 expression levels for 48 h in HK-2 cells and **C** NRK-52E cells. **B** Quantification of CD133 protein expression in HK-2 cells and **D** NRK-52E cells. **p < 0.01 versus the group at 5.5 mM concentration. **E**, **F** Western blotting analysis of CD133, Vimentin, CK-18 and PCNA expression after incubation with HG for different times (0, 6, 12, 24, and 48 h). *p < 0.05, **p < 0.01 versus the HG group at 0 h. **G** qRT-PCR analysis was carried out to compare the expression level of CD133, Vimentin and ZO-1. The two y-axes represent relative mRNA levels (left y-axis) or the fold difference in Ct value of HG over the NG group (right y-axis) in HK-2 cells and **H** NRK-52E cells. *p < 0.05, **p < 0.01 versus the group at 0 h

**Fig. 4:**
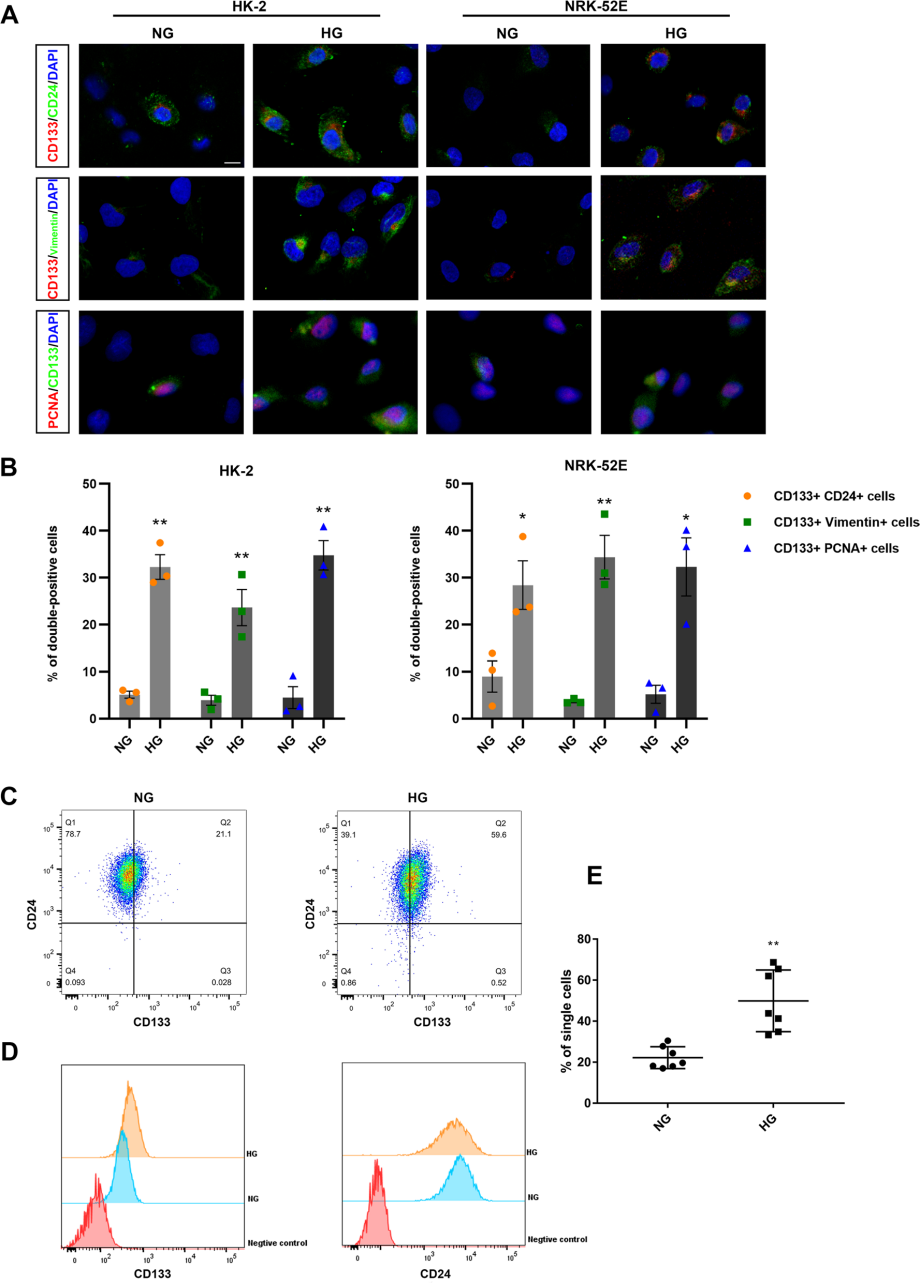
The expression patterns of CD133 in vitro. **A** Double-label immunofluorescence for CD133 and CD24 showing HG-induced renal stem/progenitor phenotype; double-label immunofluorescence for CD133 and vimentin showing HG-induced both progenitor and mesenchymal phenotype; double-label immunofluorescence for CD133 and PCNA showing the proliferative capability of CD133-positive cells. Scale bar: 10 μm. **B** Quantitative data for the ratio of double positive cells. **C**, **D** Flow cytometry analysis of CD133 and CD24 populations in HK-2 cells after HG stimulation. **E** The percentage of CD133/CD24 co-expressing cells. *p < 0.05, **p < 0.01 versus the NG group

HK-2 and NRK-52E cells were cultured for 48 h with various concentrations of glucose to determine the optimal HG concentration indicated by CD133 expression. As shown in Fig. 3A–D, CD133 expression peaked at 40 mM and 50 mM in response to HG, but too high of a concentration led to decreased expression. Therefore, final concentrations of 40 mM and 50 mM were selected to stimulate the HK-2 and NRK-52E cell lines, respectively. With increasing intervention times, the expression of CD133, Vimentin, and PCNA increased approximately threefold with HG treatment, whereas the expression of the epithelial marker CK-18 was ~ twofold reduced (Fig. 3E, F). In addition, the mRNA expression of corresponding genes was verified. A loss of the epithelial marker ZO-1 was observed, and the HG group with ~ 20% reduction compared to the control group at 48 h. The upregulation of CD133 expression seems to be more drastic in HK-2 cells, with a ~ eightfold increase compared with the NG group (Fig. 3G). The expression of CD133 was increased ~ threefold with HG treatment in NRK-52E cells (Fig. 3H). The expressions of Vimentin were upregulated approximately fourfold after HG stimulation at 48 h both in HK-2 cells and NRK-52E cells (Fig. 3G, H). To further confirm the expression pattern of CD133 in PTCs induced by HG, immunofluorescence analysis was performed in vitro. CD133/CD24 double-positive cells (CD133+/CD24+ cells) were increased ~ threefold by HG stimulation. Similarly, the coexpression of CD133 and Vimentin and the coexpression of CD133 and PCNA also increased ~ threefold after the cells were incubated with HG (Fig. 4A, B), and these were consistent with those of our in vivo experiments. Co-expression of CD133 and CD24 showed that CD24 was independently expressed, while CD133 was not, during NG intervention. We therefore performed flow cytometry to determine the expression pattern of CD133+/CD24+ cells in HK-2 cells. As expected, CD133+/CD24+ cells were markedly increased by ~ 25% after HG stimulation for 48 h and almost all HK-2 cells expressed CD24 (Fig. 4C–E). These results indicated that HG-induced increased expression of CD133 in PTCs, and the expression patterns of CD133 in vitro were similar to those in vivo.

### The protective effects of CD133 in HG-induced PTCs injury

**Fig. 5:**
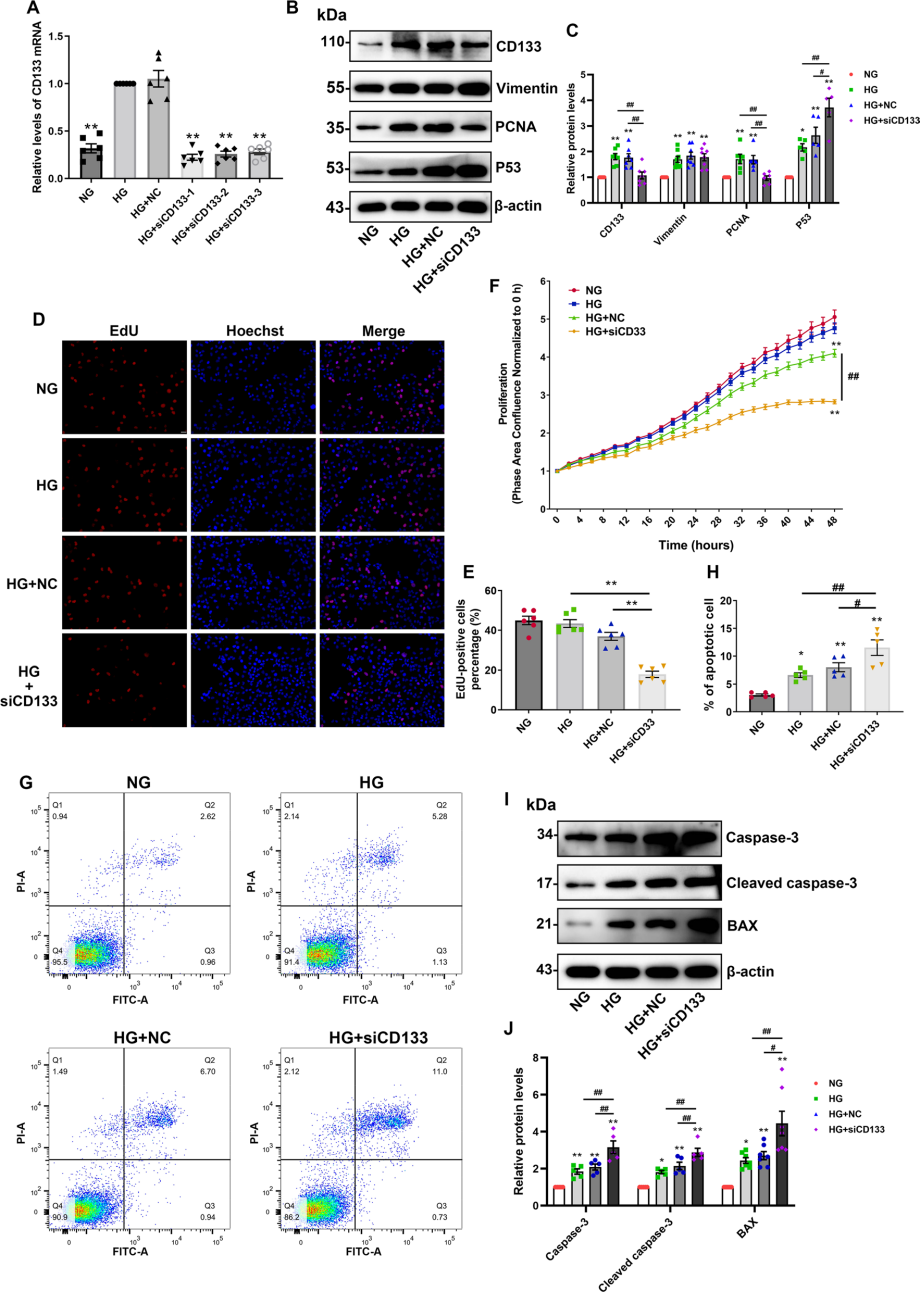
CD133-knockdown decreased cell proliferation and increased apoptosis under the HG condition in HK-2 cells. **A** the silencing effectiveness of CD133 siRNA was verified by qRT-PCR. **B**, **C** The expression of CD133, Vimentin, PCNA and P53 was detected by western blotting after CD133 knockdown. **D** EdU cell proliferation assay. The proliferation cells were double-labeled for EdU (red) and Hoechst 33342 (blue), Scale bar: 25 μm. **E** Rate of EdU-positive cells. **F** The results of real-time cell proliferation assay. **G**, **H** The apoptosis of HK-2 cells was analysed by flow cytometry. The total apoptosis rate was the sum of the Q2 and Q3 cell apoptosis ratio. **I**, **J** Western blotting analysis of Bax, caspase-3 and cleaved caspase-3 expression in the NG, HG, HG + NC and HG + siCD133 group. *p < 0.05, **p < 0.01 versus the NG group, ^#^*p* < 0.05, ^##^*p* < 0.01 versus the HG + siCD133 group

ScRNA-seq analysis indicated that genes that were coexpressed with CD133 were enriched in various biological processes, such as regulation of cell proliferation, injury repair, and cell apoptosis. We tested three small interference sequences of CD133 (siCD133-1, siCD133-2, siCD133-3); siCD133-1 had the best interference effects and was selected for subsequent experiments (Fig. 5A). The expression of CD133 and PCNA was decreased by ~ 50% when CD133 was knocked down in the presence of HG compared to that in the HG group, while the expression levels of Vimentin did not (Fig. 5B, C). Then, EdU and real-time cell proliferation assay were used to evaluate the effect of CD133 on proliferation regulation in the context of HG. The cell proliferation rate was not elevated under HG conditions, but it was significantly reduced by ~ twofold in the HG + siCD133 group (Fig. 5D–F). Additionally, these results were further validated using another siRNA (siCD133-2) (Additional file 2: Fig. S2A–D). However, CD133 overexpression showed no increase in PCNA level and cell proliferation in NG or HG condition (Additional file 3: Fig. S3A–E). In addition to proliferation, PCNA is also involved in DNA damage repair, and we conjecture that the upregulation of PCNA might be associated with the activation of damage repair processes under HG stimulation. Hence, P53, a critical upstream factor in cell repair, was examined and we found that P53 was upregulated in the HG group. Rather unexpectedly, P53 was highly expressed after the knockdown of CD133, with a ~ twofold increase compared to the HG group (Fig. 5B, C). The flow cytometry results demonstrated that the percentage of apoptotic cells was ~ 60% in the HG group, and it was  > 10% in the HG + siCD133 group (Fig. 5G, H). Moreover, the apoptotic proteins Caspase-3, cleaved caspase-3, and BAX exhibited substantial upregulation after CD133 knockdown under HG conditions (Fig. 5I, J). And, the HG + siCD133-2 group also demonstrated a noteworthy increase in Caspase-3 and BAX (Additional file 2: Fig. S2E, F). Conversely, these apoptotic proteins were significantly reduced following CD133 overexpression (Additional file 3: Fig. S3 F, G). In summary, these findings indicate that the upregulation of CD133 induced by HG may serve as a protective factor.

### Continual upregulation of CD133 might be instead involved in the pathological process of DKD

**Fig. 6:**
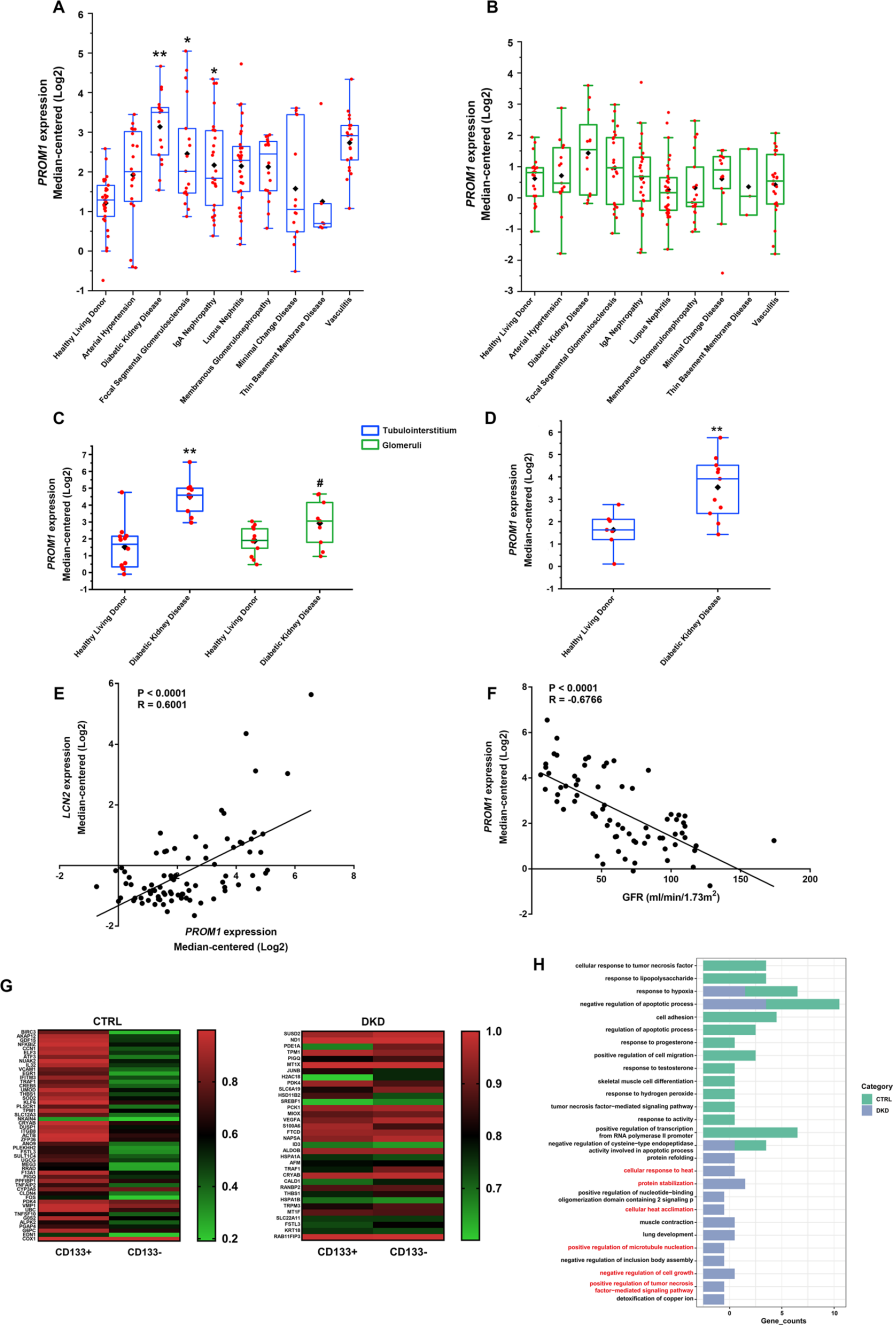
CD133 expression correlates with renal function and tubular injury. **A** The expression level of CD133 (*PROM1*) in tubulointerstitial and glomerular tissues (**B**) in DKD patients based on the study from Ju et al. **C**, **D** The expression level of CD133 (*PROM1*) in DKD patients based on the studies from Woroniecka et al. and Schmid et al., respectively. **E** Correlation analysis between NGAL (*LCN2*) and CD133 (*PROM1*), n = 84. **F** Correlation analysis between CD133 (*PROM1*) and GFR (mL/min/1.73 m^2^), n = 73. *p < 0.05, **p < 0.01 versus the healthy living donor group. **G** Heatmaps of differentially expressed genes between CD133+ cells and CD133− cells in the PTC cluster in the DKD and CTRL group, respectively. **H** GO_BP enrichment analysis of differentially expressed genes of CD133+ cells in PTC cluster

To clarify the significance of the increased expression of CD133 in DKD, the Nephroseq database (https://www.nephroseq.org/resource/login.html) was used to evaluate the expression of CD133 (*PROM1*) in human kidney biopsy samples. There were 3 datasets involved mRNA sequencing in glomerular and tubulointerstitial tissues in DKD patients [30–32]. As shown in Fig. 6A, CD133 expression was distinctly increased in tubulointerstitial tissues of DKD compared to the healthy living donor group. Furthermore, the increasing expression of CD133 was statistically significant in focal segmental glomerulosclerosis and IgA nephropathy. However, the highest CD133 expression median with ~ twofold compared to the healthy living donor group was observed in DKD, which indicated the special clinical relevance for DKD. There were no statistical differences in CD133 expression among groups of glomerular tissues (Fig. 6B). The analysis of the dataset from Woroniecka et al. [32] showed that the increased expression of CD133 in glomeruli was statistically significant. The upregulation of CD133 expression in tubulointerstitial tissues was ~ threefold in the DKD group compared to the healthy living donor group. However, the expression of CD133 was increased less than ~ 1.5-fold in glomeruli in the DKD group compared to the control group (Fig. 6C). There was no data of glomeruli in the dataset from Schmid et al. [30], and the analysis of tubulointerstitial tissues revealed a similar result (Fig. 6D). Three datasets were integrated to perform the correlations between CD133 and glomerular filtration rate (GFR) or NGAL (*LCN2*). Results showed that CD133 expression was positively correlated with the expression of NGAL (*LCN2*), and negatively correlated with GFR (mL/min.1.73 m^2^) in DKD patients (Fig. 6E, F). These results demonstrated the specificity of the increasing expression of CD133 in tubulointerstitial tissues in DKD, and the clinical relevance of CD133 in DKD progression. This is in apparent contradiction with the conclusion previous studies confirmed that CD133+ PTCs could be involved in cell repair and regeneration [8, 11–13].

Therefore, scRNA-seq analysis was further used to explore whether the property of CD133+ PTCs is altered in DKD. We reclassified the PTC cluster into CD133+ and CD133-negative (CD133−) clusters based on whether CD133 was expressed in both the control and DKD groups. The results are presented as the percentage of cells where the gene is expressed in CD133+ or CD133− cells, which can be understood as the expression level of gene in CD133+ or CD133− cells. Results showed that the differential genes between CD133+ and CD133− cells in the DKD group are different from those in the CTRL group, indicating that the characteristics of CD133+ cells are changed by DKD. Additionally, there were significant differences in the expression level of genes between the CD133+ and CD133− clusters in control samples, but the differences were reduced in the DKD group (Fig. 6G). Then, we compared these genes between the control and DKD groups and conducted GO_BP analysis. A total of 51 differential genes were enriched in 57 GO terms in the control group (Additional file 7: Table S4), and 34 differential genes were enriched in 50 GO terms in the DKD group (Additional file 8: Table S5). We display the top 15 Go terms in the bar chart (Fig. 6H). The results showed that the biological processes in which CD133+ PTCs involved are altered by DKD. CD133+ PTCs might contribute to DKD progression through various mechanisms, such as heat damage, cell microtubule stabilization, cell growth inhibition, and tumor necrosis factor-mediated signaling pathway.

## Discussion

Diabetic kidney disease (DKD) has become the leading cause of end-stage renal failure [1, 2]. The well-known RCT studies UK Prospective Diabetes Study (UKPDS) in type two diabetes [33, 34] and Diabetes Control and Complications Trial (DCCT) in type one diabetes [35] demonstrated that intensive hypoglycaemic treatment of diabetes could decrease proteinuria; however, this treatment could not prevent DKD progression to end-stage renal failure. The treatment of DKD remains an intractable clinical problem. The repair process of PTCs in acute kidney disease is incredibly encouraging, which CD133+ PTCs play an important role [8, 11–13]. However, it is unclear whether the characteristics of CD133 expression in DKD patients are similar to those in acute kidney disease. In the present study, we validated that CD133 was dominantly expressed in PTCs in DKD rats. Furthermore, we found that though the upregulation of CD133 could protect PTCs from cell apoptosis, these survival cells or CD133 itself might involved in the pathological process of DKD.

ScRNA-seq techniques allow us to measure gene expression in thousands of cells from a kidney biopsy at a single-cell resolution. Following bioinformatics analysis of the gene expression profile, the expression levels of each gene were determined in individual cells [36]. Additionally, the use of single-cell sequencing can help us to understand differentiation-driven determinants and cell fate regulation [36]. We found that CD133 was primarily expressed in the proximal and distal tubular cells and parietal epithelial cells (PECs); however, PTCs exhibited significant differences in CD133 expressing cell percentage and expression level in the DKD group compared to that in the control group. Previous studies have confirmed that CD133 is predominantly expressed in PTCs [8, 11], and some studies indicated its expression in distal tubular cells [13, 37]. PECs are defined as epithelial cells that are attached between podocytes and PTCs, and some subtypes express stem/progenitor markers, such as CD133 [38]. Interestingly, our analysis showed that CD133 was identified in the subcluster of PTCs. UMAP dimension analysis can describe the continuity of developmental timing in similar clusters, and we considered that the PTC subcluster with high expression of CD133 exhibited dual properties of PTCs and stem/progenitor cells which might be related to dedifferentiated PTCs. The expression of CD133 in healthy kidneys might indicate the self-renewal after senescence of tubular cells [8].

To better verify the expression patterns of CD133 induced by HG, a unique stimulatory factor that provokes DKD pathology, DKD rat models were established by combining STZ and unilateral nephrectomy. We found that the expression of CD133 significantly increased in the renal cortex as DKD progressed. Recent evidence confirmed that dedifferentiated PTCs participate in renal regeneration in acute kidney disease, which are usually colabeled with CD133 and CD24 [7, 8, 10]. In our study, some KIM-1 positive cells co-expressed with CD133 and CD24, suggesting that some injury PTCs might have a repair tendency. Immunofluorescence analysis showed that CD133 was also co-expressed with Vimentin, which was consistent with the results in acute kidney disease of Lindgren et al. and Smeets et al. [8, 11]. Brossa et al. indicated that CD133+ cells expressed other stem/progenitor markers SOX9 [19], and our results further corroborated the similar expression patterns of CD133. Further exploration in vitro demonstrated consistent results with those in DKD rats. It is worth noting that too high a concentration of glucose reduced CD133 expression both in HK-2 cells and NRK-52E cells. On the one hand, too high a concentration of glucose induced massive cell death, leading to the decreased expression of CD133. On the other hand, excessive high glucose tends to be acute stimulation which was validated to reduce CD133 expression [9]. The flow cytometry and immunofluorescence results indicated that CD24 was weakly expressed in all cells in vitro. Romagnani et al. reported that CD133+ cells always co-expressed with CD24 in the adult human kidney [39]. However, Shrestha et al. found CD24 was inherently expressed in three types of PTCs (HK-2, primary human proximal tubule cells, and RPTEC/TERT1 cells) [40], suggesting that the expression pattern of CD24 might be altered in vitro, but the specific mechanism requires further investigation.

Based on scRNA-seq analysis, we found that the molecular function of CD133 was associated with the following repair-related biological processes, such as cell proliferation, regulation of gene expression, and wound healing. Therefore, we tend to believe that the upregulation of CD133 expression represented the potential repair mechanism of PTCs in DKD. CD133 has been shown to promote cell proliferation in tubular repair after acute kidney injury [18, 19]. Our results showed a possibly positive relationship between CD133 and PCNA in vivo and in vitro. The coexpression of CD133 and PCNA has been mentioned in previous studies, which alluded to the proliferative and repair capability of CD133+ cells [41, 42]. However, in the present investigation, upregulation of CD133 and PCNA were observed in HK-2 cells under HG conditions, but there was no effect on cell proliferation. When CD133 was knocked down, PCNA and cell proliferation were deceased, while those were not altered when CD133 was overexpressed. PCNA plays a critical role in DNA replicative protein and DNA damage repair [43, 44]. The upregulation of PCNA expression induced by HG might be attributed to its function related to damage repair. Therefore, CD133 is not a critical upstream factor promoting cell proliferation but a protective factor in response to injury. P53 is involved in complicated biological processes; for instance, P53 is able to mediate cell proliferation and repair by promoting PCNA expression and inducing apoptosis in response to various environmental stimuli [45]. Our results showed that the expression of P53 was increased both in the HG group and the CD133 knockdown group. We speculate that HG-induced the activation of P53-mediated cell repair and apoptosis signaling pathways at the same time, and this relative balance between cell survival and death was important for maintaining cell number. However, when the protective effect of CD133 was abolished, P53-mediated apoptosis pathways were predominant, resulting in an imbalance. Additionally, CD133 has an anti-apoptotic effect in the condition of HG, although the underlying mechanism needs to further investigated. Taken together, the upregulation of CD133 expression is likely a self-protective response to early injury.

In the present study, we revealed that the expression of CD133 was gradually increased with the progress of DKD, however, this seems to contradict the protective role of CD133 in response to injury. More clinical samples are essential to clarify the significance of CD133 expression in DKD. Therefore, we used the Nephroseq database to analyse the clinical relevance of CD133 in DKD patients. We found that the increasing expression of CD133 was specific to tubulointerstitial tissues of DKD. Moreover, CD133 expression was positively correlated with NGAL* (LCN2)* and negatively correlated with GFR, implying that CD133 might to related to DKD pathology. Actually, only one previous study reported that CD133+ cells contributed to the pathology of crescentic glomerulonephritis [46]. Our result confirmed that the expression patterns of CD133 are similar to those in acute kidney disease and the protective role in response to HG-induced injury, suggesting the repair tendency of PTCs in DKD. However, this repair process is affected by the microenvironment of DKD, CD133 overexpression is instead involved in DKD pathology. ScRNA-seq analysis further revealed that the property of CD133+ PTCs is altered in DKD, and CD133+ PTCs might contribute to DKD progression through various mechanisms. Further experimental studies need to illustrate the CD133-related pathogenetic mechanism of DKD.

**Fig. 7:**
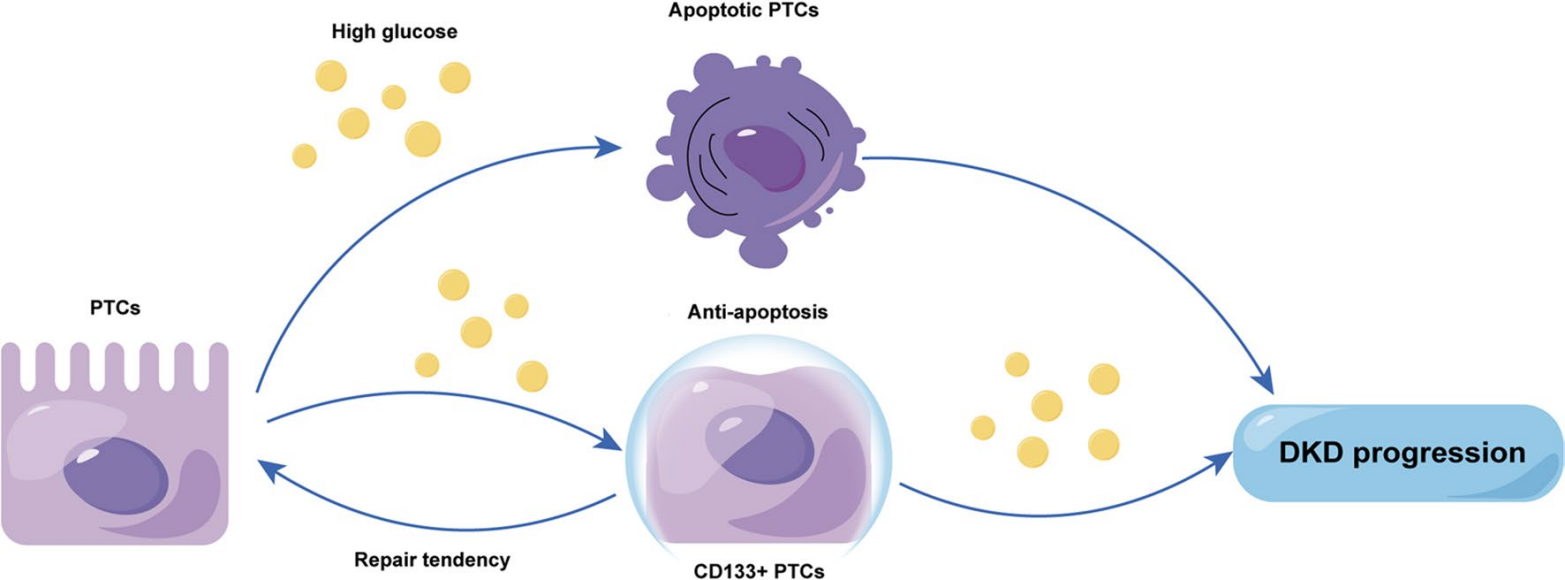
Schematic representation of the proposed hypothesis on the role of CD133+ PTCs in DKD progression. As a unique stimulatory factor to provoke DKD pathology, high glucose, could promote cell apoptosis. The increased expression of CD133 in PTCs protects cells from apoptosis, and CD133+ PTCs retain their repair tendency and could be involved in cell regeneration in certain situations. However, the vicious cycle of renal pathology in DKD makes the functions of CD133+ PTCs impaired and fails to complete the renal repair process, further aggravating kidney injury and contributing to DKD progression

Since some research reported that CD133+ cells can be involved in kidney repair in acute kidney disease, CD133 has been considered a novel therapeutic approach [47–50]. However, Burger et al. suggested that human cord blood CD133+ cells exacerbate renal injury [51]. Unexpectedly, CD133+ cells play a bad role in renal injury, and our results provide preliminary theoretical support for this surprising result. Inspired by our results, we proposed a new perspective: CD133 plays distinct roles in acute and chronic settings. At the beginning of acute injury, CD133 expression is down-regulated. However, upregulated CD133 expressions contribute to cell repair during recovery after the damage stimulus is removed. In chronic settings, though increased CD133 protect cells from apoptosis, the function of survival CD133+ cells might be altered by continuous stimuli. As a result, continually increasing expression levels of CD133 or CD133+ cells could be involved in the progression of chronic diseases, like DKD (Fig. 7). Some previous investigations provide theoretical support: the function of CD133+ cells was drastically impaired in kidney injury [52–54], and hyperglycemia exerted an effect on cell stemness [55]. More research is required to clarify the upstream regulatory mechanism, and in vivo studies need to be performed to track CD133+ PTCs in chronic settings to validate this hypothesis.

In conclusion, the present study was the first to demonstrate the role of CD133 in DKD. The anti-apoptosis capability of CD133 or CD133+ PTCs might be associated with DKD progression, which makes us revisit the regenerative potential and the pathogenicity of PTCs in DKD. A more complete understanding of the detailed mechanisms underlying CD133+ cell fate in DKD awaits future experiments.

### Supplementary Information

**Additional file 1: Figure S1.** The establishment of DKD rat models. **A**, **B** Twenty-four-hour urinary protein and microalbumin reflected impaired renal function with DKD progression. **C** Changes in the blood glucose levels in different duration groups. **D** PAS staining was used to confirm the establishment and display the early pathological changes of DKD. Glomeruli are at ×400 magnification, scale bar: 30 μm; kidney tubules are at ×200 magnification, scale bar: 50 μm. **E**, **F** Masson staining analysis. Scale bar: 50 μm. **G** Immunohistochemical analysis of NGAL. Scale bar: 100 μm. **p < 0.01 versus the Sham group; ^##^*p* < 0.01 versus the Unx group; ^&&^*p* < 0.01 versus the group of DKD at week 4; ns: indicates nonsignificant.

**Additional file 2: Figure S2.** siCD133-2 also decreased cell proliferation and increased apoptosis under the HG condition in HK-2 cells. **A**, **B** The expression of CD133, Vimentin, and PCNA was detected by western blotting after siCD133-2 intervention. **C** EdU cell proliferation assay. The proliferation cells were double-labeled for EdU (red) and Hoechst 33342 (blue), Scale bar: 25 μm. **D** Rate of EdU-positive cells. **E**,** F** Western blotting analysis of Bax and Caspase-3 expression in the NG, HG, HG + NC and HG + siCD133 group. *p < 0.05, **p < 0.01 versus the NG group, ^#^*p* < 0.05, ^##^*p* < 0.01 versus the HG + siCD133 group.

**Additional file 3: Figure S3.** Effects of CD133 overexpression on HK-2 cell proliferation and apoptosis. **A** The overexpression efficiency of CD133 plasmid was validated by qRT-PCR. **B**, **C** The expression of CD133, Vimentin and PCNA was detected by western blotting after CD133 overexpression. **D** EdU cell proliferation assay. The proliferation cells were double-labeled for EdU (red) and Hoechst 33342 (blue), Scale bar: 25 μm. **E** Rate of EdU-positive cells. **F**, **G** Western blotting analysis of Bax, caspase-3 and cleaved caspase-3 expression in the NG, HG, HG + Vector and HG + Flag-CD133 group. *p < 0.05, **p < 0.01 versus the NG group, ^#^*p* < 0.05, ^##^*p* < 0.01 versus the HG + Flag-CD133 group.

**Additional file 4: Table S1.** The top 200 genes that were positively correlated with *CD133* in the GSE131882 dataset.

**Additional file 5: Table S2.** A total of 36 GO terms enriched by 200 genes after GO_BP analysis.

**Additional file 6: Table S3.** Changes in biochemical parameters in each group.

**Additional file 7: Table S4.** A total of 57 GO terms enriched by 51 differential genes in the control group after GO_BP analysis.

**Additional file 8: Table S5.** A total of 50 GO terms enriched by 34 differential genes in the DKD group after GO_BP analysis.

## Data Availability

All data generated or analyzed during this study are available from the corresponding author on reasonable request.

## Abbreviations

BUN: Blood urea nitrogen
CD-PC: Collecting duct-principal cell
DKD: Diabetic kidney disease
DTC: Distal tubular cell
DTC/CD: Distal tubule cell/collecting duct
EMT: Epithelial–mesenchymal transition
GO_BP: GO Gene Ontology biological processes
H G: High glucose
LEUK: Leukocyte
MES: Mesangial cell
NC: Negative control
NG: Normal glucose
PEC: Parietal epithelial cell
PODO: Podocyte
PTCs: Proximal tubular cells
STC: Renal resident scattered tubular cell
SCr: Serum creatinine
scRNA-seq: Single-cell RNA sequencing
STZ: Streptozocin
