# Supplementary figures and images for: High CD133 expression in proximal tubular cells in diabetic kidney disease: good or bad?
Source: J Transl Med. 2024 Feb 16;22:159. doi: 10.1186/s12967-024-04950-0 (PMC10870558; doi:10.1186/s12967-024-04950-0)

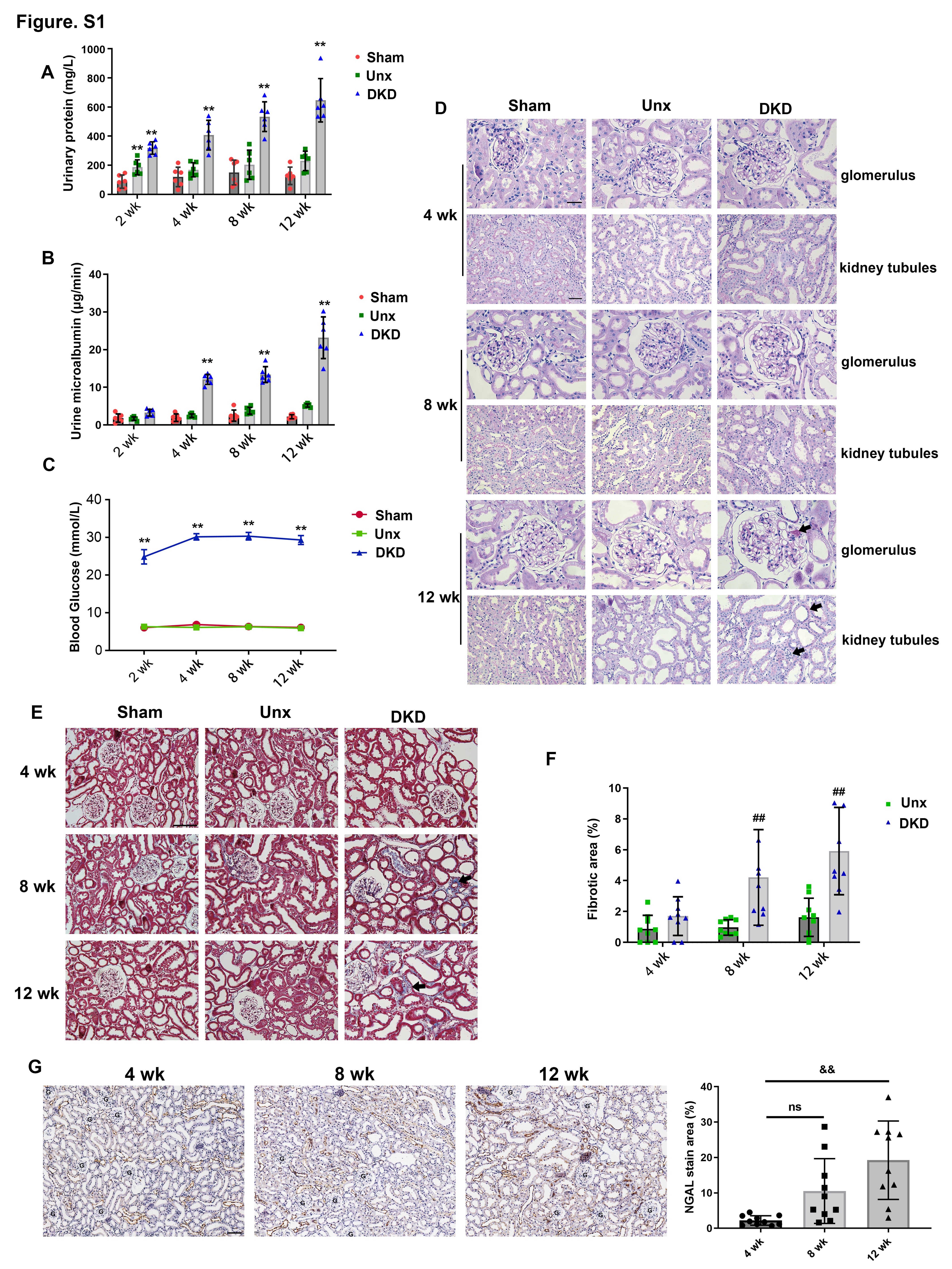

Supplement: Supplementary file 1 — Additional file 1: Figure S1. The establishment of DKD rat models. A, B Twenty-four-hour urinary protein and microalbumin reflected impaired renal function with DKD progression. C Changes in the blood glucose levels in different duration groups. D PAS staining was used to confirm the establishment and display the early pathological changes of DKD. Glomeruli are at ×400 magnification, scale bar: 30 μm; kidney tubules are at ×200 magnification, scale bar: 50 μm. E, F Masson staining analysis. Scale bar: 50 μm. G Immunohistochemical analysis of NGAL. Scale bar: 100 μm. **p < 0.01 versus the Sham group; ##p < 0.01 versus the Unx group; &&p < 0.01 versus the group of DKD at week 4; ns: indicates nonsignificant. [file 12967_2024_4950_MOESM1_ESM.tif]

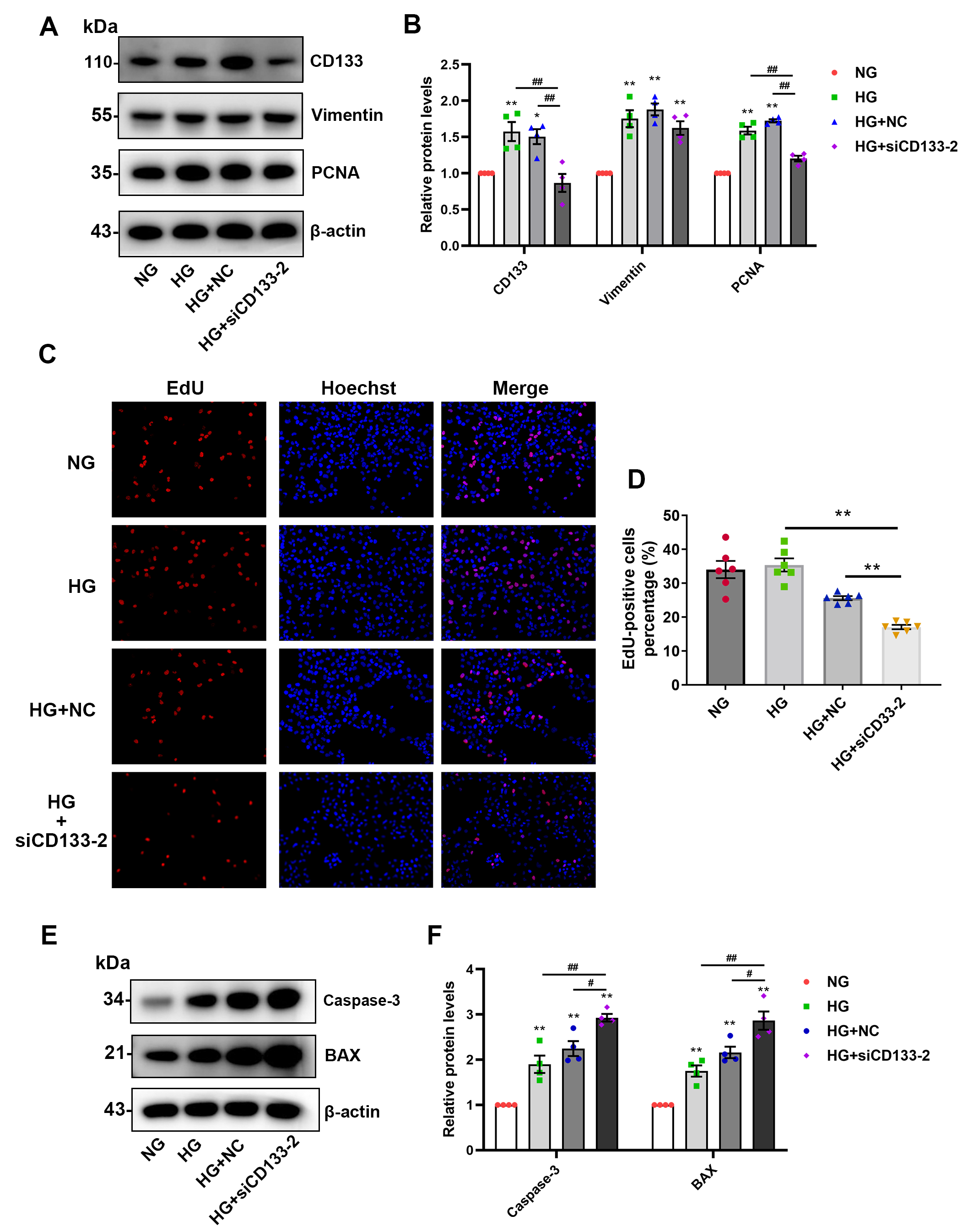

Supplement: Supplementary file 2 — Additional file 2: Figure S2. siCD133-2 also decreased cell proliferation and increased apoptosis under the HG condition in HK-2 cells. A, B The expression of CD133, Vimentin, and PCNA was detected by western blotting after siCD133-2 intervention. C EdU cell proliferation assay. The proliferation cells were double-labeled for EdU (red) and Hoechst 33342 (blue), Scale bar: 25 μm. D Rate of EdU-positive cells. E, F Western blotting analysis of Bax and Caspase-3 expression in the NG, HG, HG + NC and HG + siCD133 group. *p < 0.05, **p < 0.01 versus the NG group, #p < 0.05, ##p < 0.01 versus the HG + siCD133 group. [file 12967_2024_4950_MOESM2_ESM.tif]

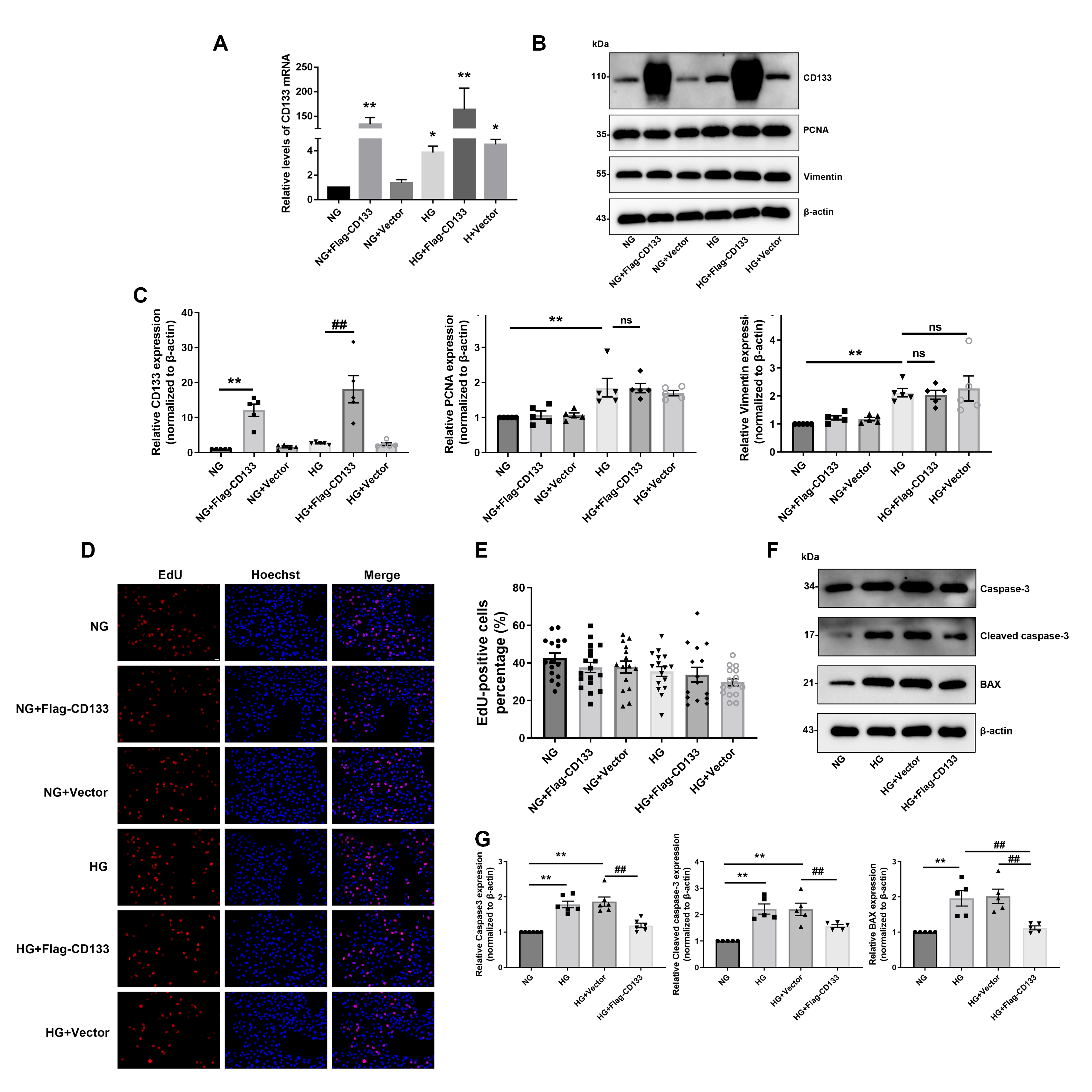

Supplement: Supplementary file 3 — Additional file 3: Figure S3. Effects of CD133 overexpression on HK-2 cell proliferation and apoptosis. A The overexpression efficiency of CD133 plasmid was validated by qRT-PCR. B, C The expression of CD133, Vimentin and PCNA was detected by western blotting after CD133 overexpression. D EdU cell proliferation assay. The proliferation cells were double-labeled for EdU (red) and Hoechst 33342 (blue), Scale bar: 25 μm. E Rate of EdU-positive cells. F, G Western blotting analysis of Bax, caspase-3 and cleaved caspase-3 expression in the NG, HG, HG + Vector and HG + Flag-CD133 group. *p < 0.05, **p < 0.01 versus the NG group, #p < 0.05, ##p < 0.01 versus the HG + Flag-CD133 group. [file 12967_2024_4950_MOESM3_ESM.tif]
