# Supplementary material for: High CD133 expression in proximal tubular cells in diabetic kidney disease: good or bad?
Source: J Transl Med. 2024 Feb 16;22:159. doi: 10.1186/s12967-024-04950-0 (PMC10870558; doi:10.1186/s12967-024-04950-0)
Supplement: Supplementary file 6 — Additional file 6: Table S3. Changes in biochemical parameters in each group. [file 12967_2024_4950_MOESM6_ESM.docx]

**Table S3** Changes in biochemical parameters in each group.

|  |  | **Body weight (g)** | **K/W (mg/g)** | **BUN (mg/dl)** | **Scr (μmol/L)** |
| --- | --- | --- | --- | --- | --- |
| **4 wk** | **Sham** | 424.8±24.13 | 3.17±0.24 | 19.70±1.77 | 26.30±3.06 |
|  | **Unx** | 448.8±2.98 | 4.66±0.19 | 22.39±3.10 | 63.20±6.97 |
|  | **DKD** | 352.3±3.59^**^ | 7.75±0.69^*^ | 29.18±2.34^*^ | 119.54±7.20^**^ |
| **8 wk** | **Sham** | 450.1±4.42 | 3.47±0.22 | 20.12±1.80 | 52.33±5.19 |
|  | **Unx** | 486±5.78 | 4.97±0.15^*^ | 27.12±1.42 | 67.34±4.48 |
|  | **DKD** | 334.8±5.00^**^ | 8.29±0.66^**^ | 34.55±2.23^*^ | 350.80±4.85^*^ |
| **12 wk** | **Sham** | 495.5±6.20 | 3.78±0.03 | 18.59±0.25 | 69.12±0.63 |
|  | **Unx** | 542.5±5.76 | 5.26±0.88 | 27.30±0.84^*^ | 92.42±7.04 |
|  | **DKD** | 408.2±5.92^**^ | 8.77±0.75^**^ | 40.89±1.19 ^**^ | 684.16±6.77^*^ |

**K/W**: the kidney/body weight ratio; **BUN**: blood urea nitrogen; **Scr**: serum creatinine. n= 6-10 per group. *p < 0.05, **p < 0.01 versus the Sham group in respective course of disease.
